# Downregulation of RIP3 ameliorates the left ventricular mechanics and function after myocardial infarction *via* modulating NF-κB/NLRP3 pathway

**DOI:** 10.1515/biol-2022-0890

**Published:** 2024-06-18

**Authors:** Han Zhang, Yuan Yin, Shan Chen, Peipei Qian, Ganglin Zou, Yumei Liu, Junying Yang, Haining Zhang

**Affiliations:** Department of Stomatology, The First Affiliated Hospital, Sun Yat-sen University, Guangzhou 510080, China; Key Laboratory of Molecular Target & Clinical Pharmacology and The State & NMPA Key Laboratory of Respiratory Disease, School of Pharmaceutical Sciences & The Fifth Affiliated Hospital, Guangzhou Medical University, Guangzhou, 511436, P.R. China; Department of Pharmacy, Affiliated Guangxi International Zhuang Medical Hospital, Guangxi University of Traditional Chinese Medicine, Guangxi, 530021, P.R. China; Nanhai Mental Health Center, People's Hospital of Nanhai District, Foshan, 528200, P.R. China; Department of Pharmacology, Jiaying University, Meizhou, 514031, P.R. China; Department of Pharmacology, Guangzhou Medical University, Guangzhou, 511436, China

**Keywords:** RIP3, NF-κB, NLRP3, left ventricular mechanics, myocardial infarction

## Abstract

Adverse cardiac mechanical remodeling is critical for the progression of heart failure following myocardial infarction (MI). We previously demonstrated the involvement of RIP3-mediated necroptosis in the loss of functional cardiomyocytes and cardiac dysfunction post-MI. Herein, we investigated the role of RIP3 in NOD-like receptor protein 3 (NLRP3)-mediated inflammation and evaluated the effects of RIP3 knockdown on myocardial mechanics and functional changes after MI. Our findings revealed that mice with MI for 4 weeks exhibited impaired left ventricular (LV) myocardial mechanics, as evidenced by a significant decrease in strain and strain rate in each segment of the LV wall during both systole and diastole. However, RIP3 knockdown ameliorated cardiac dysfunction by improving LV myocardial mechanics not only in the anterior wall but also in other remote nonischemic segments of the LV wall. Mechanistically, knockdown of RIP3 effectively inhibited the activation of the nuclear factor kappa-B (NF-κB)/NLRP3 pathway, reduced the levels of interleukin-1β (IL-1β) and interleukin-18 (IL-18) in the heart tissues, and mitigated adverse cardiac remodeling following MI. These results suggest that downregulation of RIP3 holds promise for preventing myocardial inflammation and cardiac mechanical remodeling following MI by regulating the NF-κB/NLRP3 pathway.

## 1 Introduction

Despite significant advancements in the treatment of coronary artery disease, myocardial infarction (MI) remains a leading cause of morbidity and mortality worldwide [1]. Following MI, maladaptive cardiac remodeling with changes in cardiac mechanics contributes to the deterioration of cardiac performance and the progression to heart failure. Pathologically, the loss of functional cardiomyocytes due to cell death and the excessive activation of the inflammatory response after MI are major contributors to this process [2,3]. Therefore, preventing inflammation and cardiomyocyte death following MI could be an effective therapeutic strategy to ameliorate cardiac remodeling and delay the onset of heart failure.

Necroptosis, a caspase-independent programmed necrosis, is typically considered as a highly proinflammatory mode of cell death, which has been shown to play a significant role in the loss of cardiomyocytes following MI [3,4]. As a key mediator of necroptosis, RIP3 has been implicated in the pathogenesis of neurodegenerative diseases, inflammation, and post-ischemic cardiac dysfunction [5,6,7,8,9,10,11]. It has been reported that significantly higher plasma concentrations of RIP3 are observed in patients with heart failure, which is critically related to the prognosis of heart failure [12]. Our previous research showed that RIP3 is progressively upregulated in the infarct border zone of the hearts in MI mice. Knockdown of RIP3 improved the death of necrotic cardiomyocytes, reduced MI size, and significantly ameliorated MI-induced cardiac dysfunction [13]. However, the inflammatory mechanisms underlying RIP3 and the impact of RIP3 knockdown on left ventricular (LV) myocardial mechanics and function following MI are still not fully understood.

Recent studies have highlighted the activation of the NOD-like receptor protein 3 (NLRP3) inflammasome in the ischemic heart. NLRP3 inflammasome and NLRP3 inflammasome-related proinflammatory cytokines such as interleukin-1β (IL-1β) and interleukin-18 (IL-18) have been observed in the periphery plasma of patients with MI [14,15]. Inhibition of the NLRP3 inflammasome has been shown to reduce the inflammatory response and improve cardiac remodeling and cardiac dysfunction in animal models of acute MI [16,17,18]. Additionally, the Canakinumab Antiinflammatory Thrombosis Outcome Study trial demonstrated the efficacy of IL-1β inhibition in secondary prevention of cardiovascular events in patients with a history of MI [19], suggesting that targeting NLRP3-mediated inflammation may be a promising strategy for MI treatment.

Strain analysis using speckle tracking echocardiography (STE) has emerged as an effective tool for detecting changes of both global and regional of LV mechanics and function during cardiac remodeling in various cardiac pathologies. In comparison with the limited sensitivity of traditional echocardiographic indicators of systolic–diastolic dysfunction, STE is less angle-dependent and offers high accessibility and reproducibility among different operators [20,21]. Additionally, reduced LV myocardial deformation (strain) has been shown to correlate with the extent of MI and can serve as an independent predictor of poor prognosis in heart failure [22,23,24].

In this study, we aimed to investigate the role of RIP3 in NLRP3-mediated inflammation and evaluate the effects of RIP3 knockdown on LV mechanical and functional alterations following MI.

## 2 Materials and methods

### 2.1 Animal model and treatment

C57BL/6 male mice weighing 22–25 g were obtained from the experimental animal center of Guangdong Province in Guangzhou, China. The mice were provided with a standard diet and had unrestricted access to water. They were cared for in compliance with the Eighth Edition of the Guide for *the Care and Use of Laboratory Animals* (2011, published by The National Academies Press). The study was conducted in accordance with the Basic and Clinical Pharmacology and Toxicology policy for experimental and clinical studies [25].

MI was induced by permanently ligating the left anterior descending (LAD) coronary artery. Briefly, mice were anesthetized with sodium pentobarbital (50 mg/kg, intraperitoneal injection) and artificially ventilated using an animal ventilator (DH-140, Zhejiang, China). A thoracotomy was performed at the third or fourth intercostal space, and the LAD coronary artery was ligated. Mice in the sham control group underwent the same surgical procedure without ligating the LAD coronary artery.

A total of 24 mice were randomly divided into four groups, with equal numbers in each group: (i) sham group, (ii) MI group, (iii) Scramble shRNA+MI group, and (iv) RIP3 shRNA+MI group. Mice in the scramble shRNA+MI group or RIP3 shRNA+MI group received a cardiac-specific gene delivery of lentivirus carrying scramble shRNA (2 × 10^7^ PFU) or RIP3 shRNA (2 × 10^7^ PFU) 3 days before MI. 4 weeks after MI, mice were euthanized by inhalation of isoflurane (flow rate: 300–500 ml/min, anesthesia concentration: 1–1.5%), followed by cervical dislocation. The hearts were then harvested and processed for histological or western blotting analysis.


**Ethical approval:** The research related to animal use has been complied with all the relevant national regulations and institutional policies for the care and use of animals, and has been approved by the Institutional Animal Care and Use Committee of Guangzhou Medical University (approval number: G2022-212).

### 2.2 Generation of RIP3 shRNA construct and lentiviral preparation

Following the manufacturer’s instructions, shRNA against RIP3 was inserted into the pLKO.1 lentiviral vector (Open Biosystems, Ottawa, Canada). The construct was confirmed by DNA sequence analysis. Lentivirus expressing scramble shRNA or RIP3 shRNA was prepared by co-transfecting scramble shRNA or RIP3 shRNA lentiviral plasmids with a packaging plasmid into HEK-293T cells using FuGENE6 reagent (Roche, Indianapolis, IN, USA).

### 2.3 *In vivo* cardiac-specific gene manipulation by intramyocardial injection in mice

As previously described [13], *in vivo* cardiac-specific gene delivery was performed. The mouse heart was quickly exposed under anesthesia at the fifth intercostal space. Lentivirus carrying scramble shRNA (2 × 10^7^ PFU) or RIP3 shRNA (2 × 10^7^ PFU) was delivered into the LV free wall through three separate intramyocardial injections, respectively. Knockdown of RIP3 was confirmed by western blotting.

### 2.4 Echocardiography

Cardiac function was assessed before and 4 weeks after MI using the Vevo 2100, a high-resolution imaging system equipped with a 25 MHz imaging transducer (Vevo 2100; VisualSonics Inc., Ontario, Canada). Two-dimensional echocardiograms were obtained from the apical four chambers and parasternal long- and short-axis views. Cardiac function parameters, including LV anterior wall in diastole and LV anterior wall in systole, were measured using the Vevo 2100. The modified biplane Simpson’s method was employed to calculate the left ventricular ejection fraction (LVEF) and left ventricular fractional shortening (LVFS). Additionally, according to the instructions, other parameters such as the ratio of E-wave velocity to A-wave velocity (E/A), isovolumetric relaxation time (IVRT) and isovolumetric contraction time (IVCT), and early diastolic mitral annulus velocity/late diastolic mitral annulus velocity (E′/A′) were also analyzed.

### 2.5 Speckle tracking analysis

The high frame rate 2D images of the LV short-axis views were obtained by recording three consecutive heart cycles with the Vevo 2100. The LV endocardial border was automatically traced and manually adjusted as needed using offline software (EchoPac 201, General Electric Vingmed) that divided the short-axis view of the LV into six segments (anterior wall, interventricular septum, posterior wall, inferior wall, inferior ventricular septum, and lateral wall). Strain analysis was performed on each myocardial segment to determine the peak-systolic radial strain (Srad-S), peak-systolic circumferential strain (Scir-S), systolic peak of radial strain rate (SRrad-S), systolic peak of circumferential strain rate (SRcir-S), peak value in early diastole peak (SRrad-E, SRcir-E), and late diastolic peak (SRrad-A, SRcir-A). The percentage decrease of SRrad and SRcir in each segment was calculated according to the formula:

\[{[}(\text{Myocardial strain rate before myocardial infarction}-\text{Myocardial strain rate after myocardial infarction})/\text{Myocardial strain rate before myocardial infarction}]\times \text{}100 \% .]\]

### 2.6 Histologic analysis

Mice were euthanized, and their heart tissues were collected. The heart tissue blocks were fixed in 4% paraformaldehyde, embedded in paraffin, and then sectioned. After routine dewaxing, hematoxylin and eosin staining and Masson trichrome staining were performed. The microscopic findings were recorded using appropriate objective lenses. Fibrosis in the LV was determined by measuring the area of fibrotic tissue (stained blue for collagen) over the LV area (excluding background) using ImageJ software (NIH, version 1.30, http://rsb.info.nih.gov/ij/). The researcher, who was blinded to the treatments, examined more than five fields in three different sections for each mouse.

### 2.7 Lactate dehydrogenase (LDH) assay

The proteins from the heart tissues were extracted using RIPA lysis buffer and then centrifuged at 3,000 rpm for 10 min. The levels of LDH in supernatant were measured using a commercially available kit (Jian Cheng Institute of Biotechnology in Nanjing, China) following the instructions provided by the manufacturer.

### 2.8 mRNA isolation and quantitative real-time polymerase chain reaction (RT-qPCR)

TRIzol reagent (TaKaRa Biomedical Technology, Beijing, Co., Ltd) was used to extract total RNA. An equal amount of purified RNA was then reverse-transcribed using the RNA PCR Kit following the manufacturer’s instructions. The resulting cDNAs were amplified, and real-time PCR was conducted using primers specific for alpha-smooth muscle actin (α-SMA) and collagen I (Life technology, Invitrogen, Ltd. Paisley PA4 9RF, UK). The mRNA levels of genes were normalized to the β-actin mRNA level.

### 2.9 Cytokine assay

The proteins from the heart tissues were extracted using RIPA lysis buffer, and the protein content was quantified by the bicinchoninic acid (BCA) protein assay (Pierce). The amount of IL-1β and IL-18 in supernatants was measured using enzyme linked immunosorbent assay (ELISA) kits (eBioScience, San Diego, California, USA) following the manufacturer’s instructions.

### 2.10 Western blotting analysis

The proteins were extracted from heart tissues, and the protein concentrations were determined by BCA Protein Assay Reagent Kit (Pierce). Equal amount of proteins was separated by sodium dodecyl sulfate-polyacrylamide gel electrophoresis (SDS-PAGE) and transferred to polyvinylidene fluoride (PVDF) membranes (Roche Molecular Biochemicals, Mannheim, Germany). The membranes were blocked and subsequently probed with anti-RIP3 antibody (1:1,000, Cat# BS7363), anti-NLRP3 antibody (1:1,000, Cat# BS66103), anti-caspase 1 (p20) antibody (1:1000, Cat# BS7071), anti-β-actin antibody (1:8,000, Cat# AP0731, Bioworld Technology, St. Louis Park, MN, USA), and antibodies against p65 (1:1,000, Cat #8242) and p-p65 (Ser536) (1:1,000, Cat #3033), Cell Signaling Technology, Boston, USA). The density of the target bands was accurately quantified using the computer-aided Quantity One analysis system. β-actin was used as a loading control.

### 2.11 Statistical analysis

All data were expressed as mean ± standard deviation. Differences between two groups were assessed using the Student *t*-test performed with GraphPad Prism 8.0 software (GraphPad Software Inc). Differences between two or more groups were assessed using a one-way analysis of variance (ANOVA) followed by the Tukey post hoc test. Correlations were determined by conducting linear regression analyses. Statistical significance was defined as a *P* value of 0.05 or less.

## 3 Results

### 3.1 RIP3 knockdown preserved cardiac function post-MI

**Figure 1:**
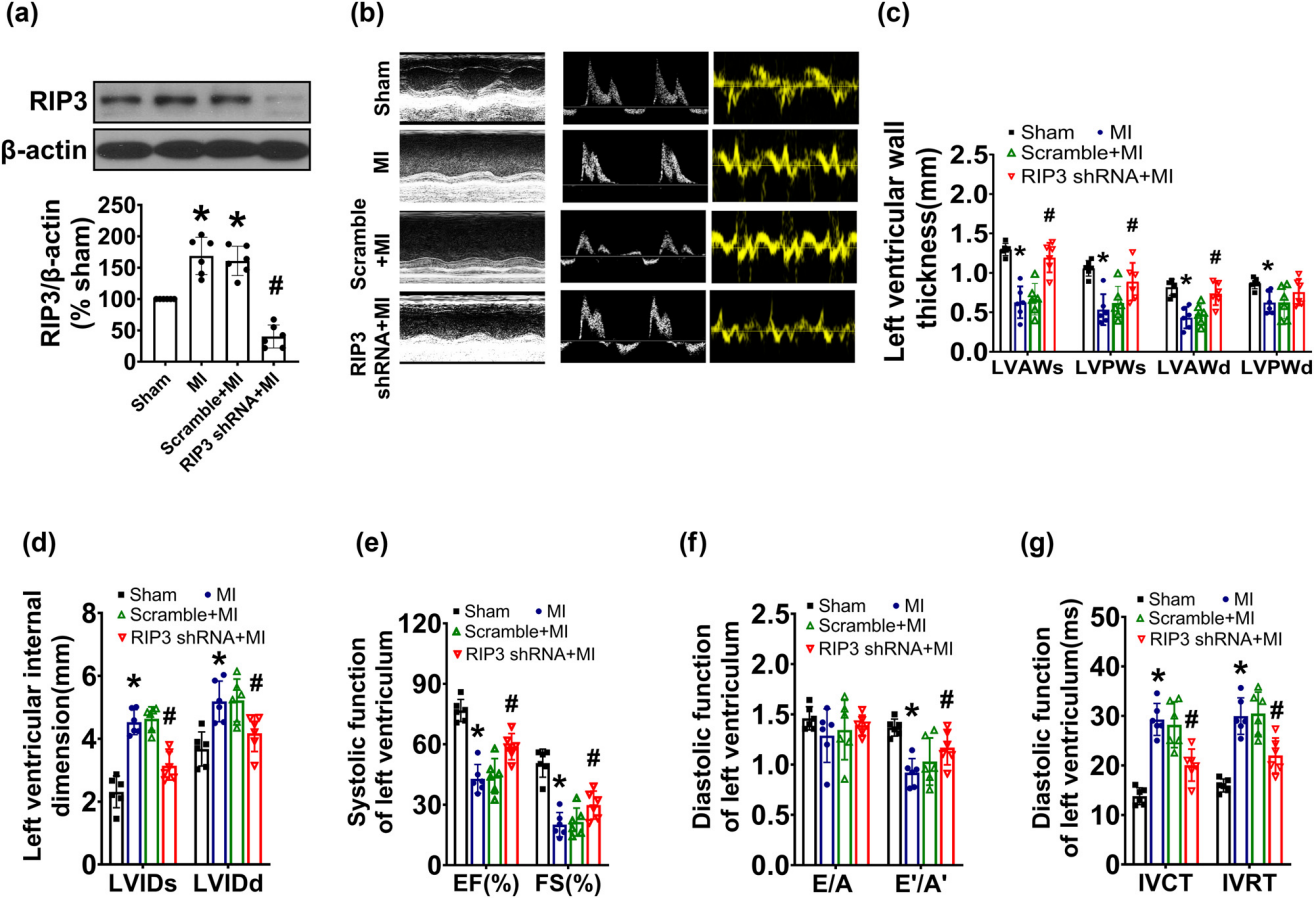
RIP3 knockdown ameliorated cardiac dysfunction after MI. Cardiac-specific gene knockdown was performed through *in vivo* intramyocardial delivery of lentivirus-encoded RIP3 shRNA 3 days prior to MI. Cardiac function was assessed by echocardiograms 4 weeks after MI: (a) the expression of RIP3 was determined by western blotting after MI (one-way ANOVA, *n* = 6, *P < 0.05 vs sham, ^#^ *P* < 0.05 vs MI); (b) representative M-mode echocardiograms and transmitral flow and tissue Doppler echocardiograms; and (c)–(g) the analyzed results of cardiac function obtained from mice in each experimental group (one-way ANOVA, *n* = 6, *P < 0.05 vs sham, ^#^ *P* < 0.05 vs MI).

Lentivirus-encoded RIP3 shRNA was delivered intramyocardially to specifically target the cardiac tissue in mice. 3 days after the RIP3 knockdown, a permanent ligation of the LAD coronary artery was performed to induce MI. As shown in Figure 1, protein expression of RIP3 in heart tissue increased in MI mice compared to sham mice. The targeted shRNA against RIP3 successfully achieved a significant cardiac-specific knockdown of RIP3 protein. In contrast, scramble lentiviruses had no effect on the expression of RIP3 protein. 4 weeks after MI, the left ventricular anterior wall (LVAW) and left ventricular posterior wall (LVPW) exhibited thinning, and the left ventricular internal dimension (LVID) at both the end of diastole and the end of systole enlarged in MI mice compared to the sham-operated mice. The fractional shortening and ejection fraction (EF) of the LV were also significantly reduced in MI mice. Echocardiography analysis showed no significant difference in *E*/*A* among the sham mice, MI mice, mice with scramble shRNA and mice with RIP3 knockdown. However, both the IVRT and IVCT were prolonged, and the E′/A′ was significantly decreased in the MI mice, indicating impaired cardiac systolic and diastolic function after MI. Compared to MI mice, intramyocardial delivery of lentivirus-encoded scramble sequence had no effect on cardiac dysfunction induced by MI. However, mice with RIP3 knockdown showed significant improvements in both cardiac systolic and diastolic functions, as evidenced by increased LVEF, LVFS, as well as LVAW and LVPW. Additionally, after RIP3 knockdown, LVID was decreased, and the prolonged IVRT and IVCT, as well as decreased E′/A′ ratio, was all improved. Overall, these findings indicate that RIP3 knockdown can ameliorate the contractile and diastolic dysfunction observed 4 weeks post-MI.

### 3.2 RIP3 knockdown mitigated cardiac mechanics after MI

**Figure 2:**
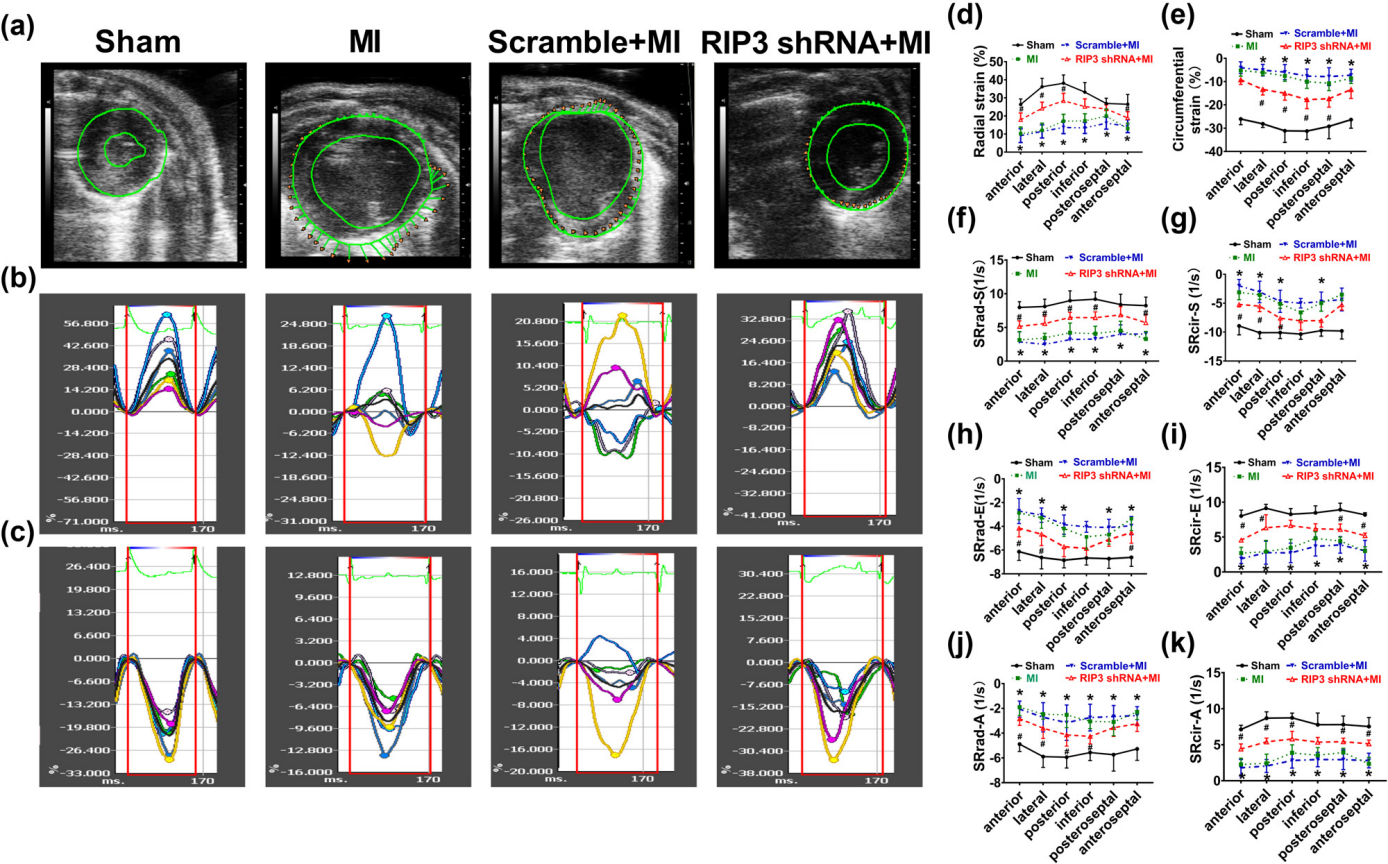
RIP3 knockdown mitigated cardiac mechanics after MI: (a) LV strain was measured by speckle-tracking imaging from the midventricular short-axis view; (b) representative radial strain curves; (c) circumferential strain curves; (d) and (e) distribution of radial and circumferential strains in sham mice, MI mice, Scramble shRNA+MI mice and mice with RIP3 knockdown (Student’s *t* test, *n* = 6, *P < 0.05 vs sham, ^#^ *P* < 0.05 vs MI); (f) and (g) distribution of radial and circumferential systolic strain rates in sham mice, MI mice, Scramble shRNA+MI mice, and mice with RIP3 knockdown (Student’s *t* test, *n* = 6, *P < 0.05 vs sham, ^#^ *P* < 0.05 vs MI); (h) and (i) distribution of radial and circumferential early-diastolic strain rates in sham mice, MI mice, Scramble shRNA+MI mice, and mice with RIP3 knockdown (Student’s *t* test, *n* = 6, *P < 0.05 vs sham, ^#^ *P* < 0.05 vs MI); and (j) and (k) distribution of radial and circumferential late-diastolic strain rates in sham mice, MI mice, Scramble shRNA+MI mice, and mice with RIP3 knockdown (Student’s *t* test, *n* = 6, *P < 0.05 vs sham, ^#^ *P* < 0.05 vs MI).

**Figure 3:**
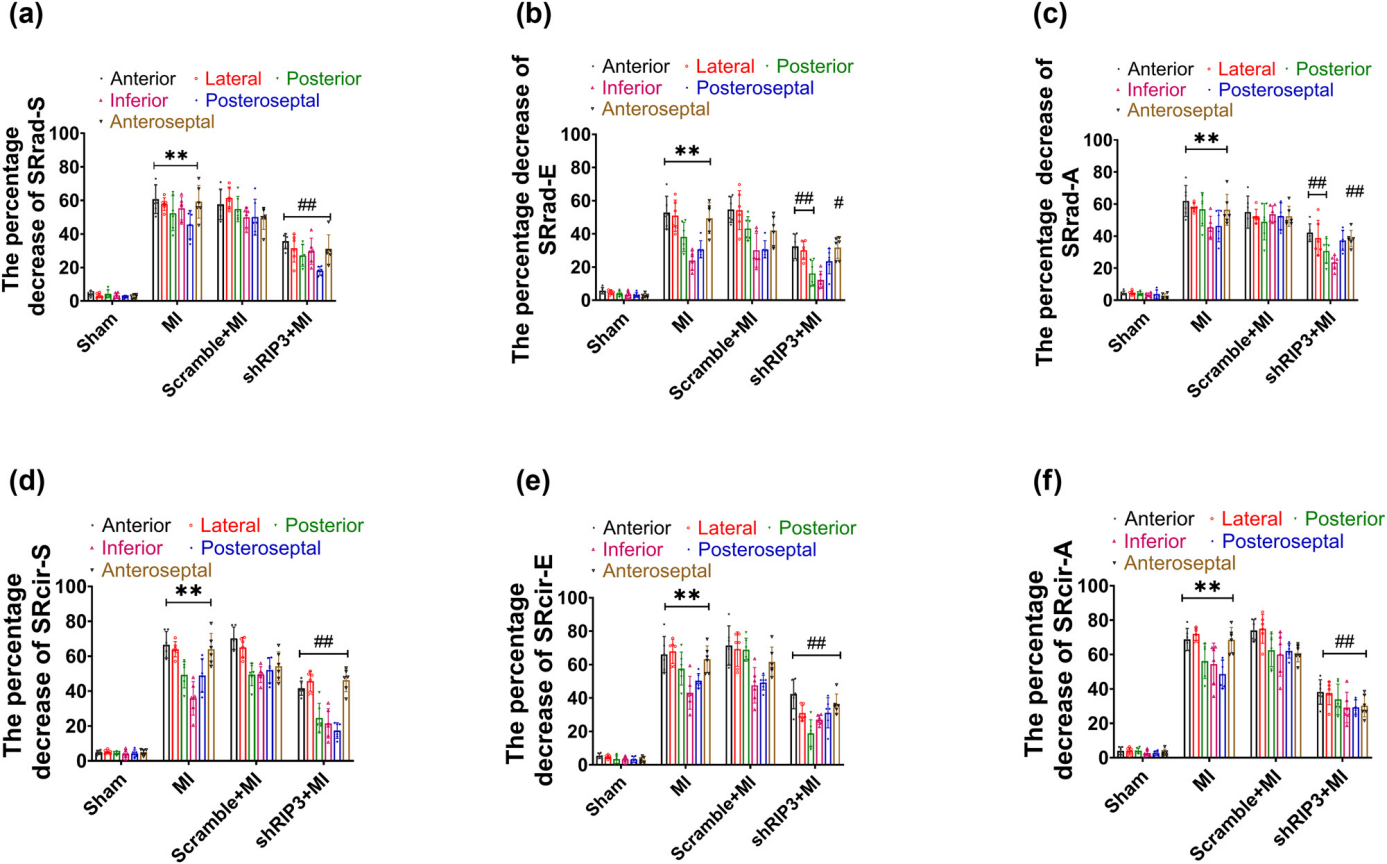
RIP3 knockdown lowered the percentage decrease of stain rate: (a)–(c) RIP3 knockdown decreased the percentage decrease in SRrad-S, SRrad-E, and SRrad-A (one-way ANOVA, *n* = 6, **P < 0.01 vs Sham, ^#^ *P* < 0.05 or ^##^ *P* < 0.01 vs MI) and (d)–(f) RIP3 knockdown decreased the percentage decrease in SRcir-S, SRcir-E, and SRcir-A (one-way ANOVA, *n* = 6, **P < 0.01 vs Sham, ^##^ *P* < 0.01 vs MI).

Speckle tracking analysis was performed to evaluate LV mechanical function after MI. Compared to sham mice, the peak value of the Srad-S, Scir-S, SRrad-S, SRcir-S, and peak value in early diastole of SRrad or SRcir (SRrad-E, SRcir-E) and late diastolic peak value of SRrad or SRcir (SRrad-A, SRcir-A) were all significantly lower in MI mice, mice delivered intramyocardially with scramble sequence, and mice with RIP3 knockdown. These decreases were observed in almost all segments (Figure 2). Notably, the anterior and lateral wall myocardium exhibited the greatest reduction in the percentage of peak-systolic radial strain and radial strain rate. None of these parameters were affected by the intramyocardial delivery of lentivirus-encoded scramble sequence compared to MI mice. However, the percentage decrease of SRrad-S, SRcir-S, SRrad-E, SRcir-E, SRrad-A, and SRcir-A in mice with RIP3 knockdown was significantly less pronounced (Figure 3).

### 3.3 Cardiac performance was negatively correlated with the decrease percentage of strain rate

**Figure 4:**
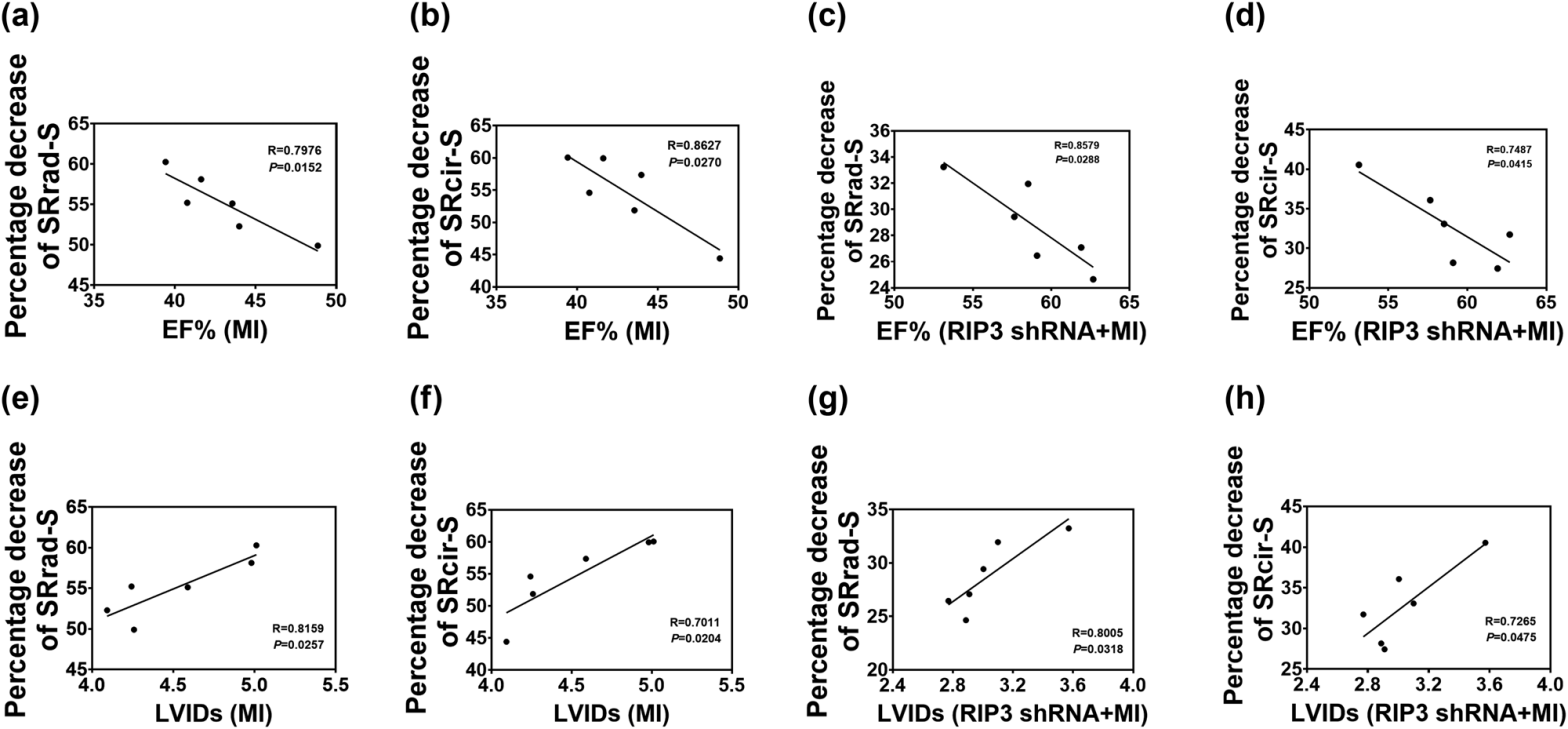
Correlations between the average percentage decrease in strain rate and LVEF and LVIDs were examined: (a)–(d) correlations between the average percentage decrease in SRrad-S and SRcir-S and LVEF in both MI mice and mice with RIP3 knockdown and (e)–(h) correlations between the average percentage decrease in SRrad-S and SRcir-S and LVIDs in both MI mice and mice with RIP3 knockdown.

After analyzing the segmental data, a strong negative correlation between the average percentage decrease in SRrad-S and SRcir-S and LVEF in both MI mice and mice with RIP3 knockdown was observed. The correlation coefficients (*r*) were 0.897 and 0.862 in MI mice and 0.857 and 0.828 in mice with RIP3 knockdown, respectively. Conversely, the average percentage decrease in SRrad-S and SRcir-S positively correlated with LV end-systolic diameter (LVIDs), with correlation coefficients (*r*) of 0.866 and 0.881 in MI mice and 0.85 and 0.816 in mice with RIP3 knockdown, respectively (Figure 4).

### 3.4 RIP3 knockdown mitigated adverse cardiac remodeling induced by MI

**Figure 5:**
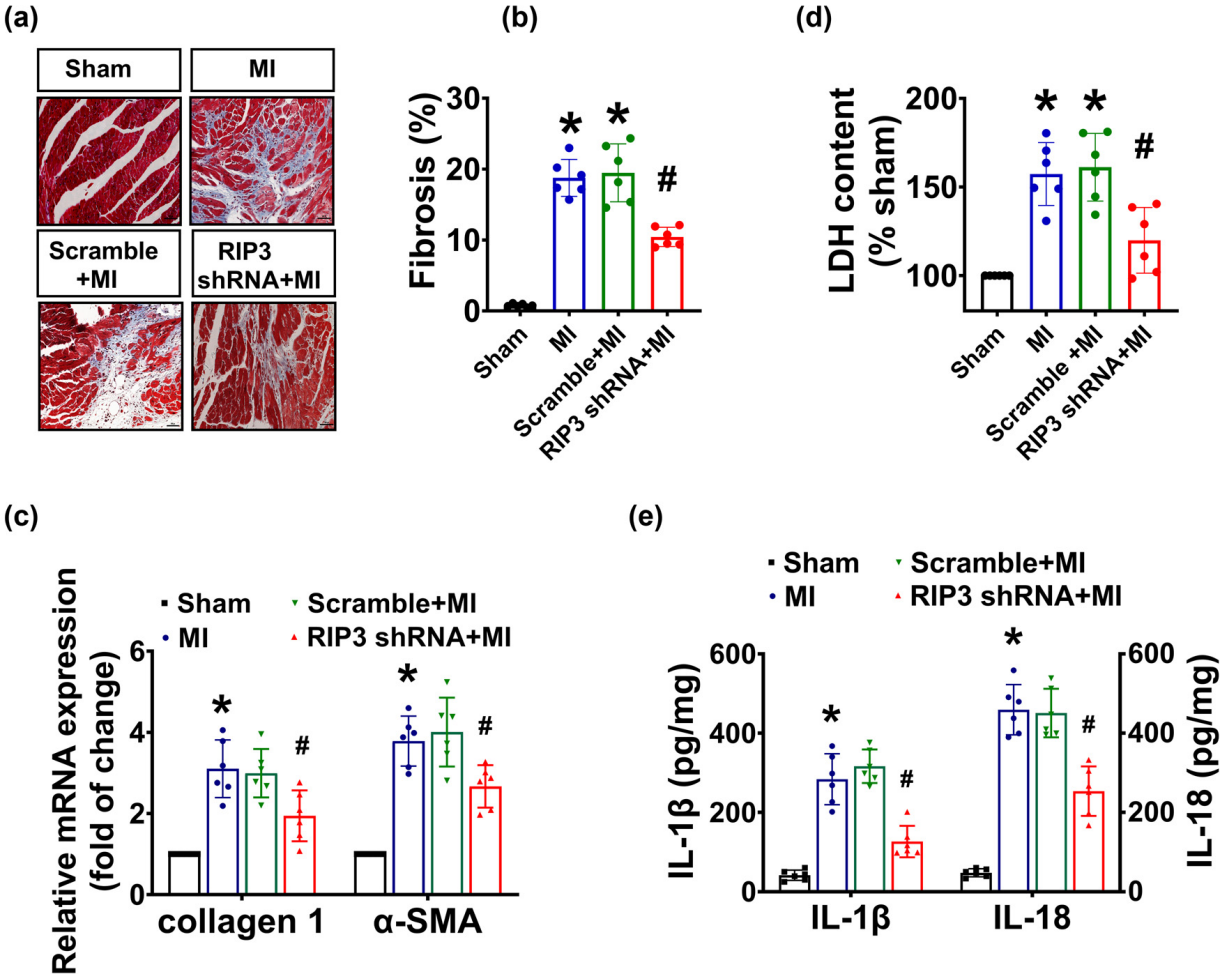
RIP3 knockdown ameliorated cardiac remodeling after MI. Cardiac-specific gene knockdown was performed through *in vivo* intramyocardial delivery of lentivirus-encoded RIP3 shRNA 3 days prior to MI. The heart was removed 4 weeks after MI: (a) representative cross-sectional images showed Masson staining; (b) analysis results for Masson staining (one-way ANOVA, *n* = 6, *P < 0.05 vs sham, ^#^ *P* < 0.05 vs MI); (c) mRNA levels of α-SMA and collagen I in the heart were determined by real-time RCR (one-way ANOVA, *n* = 6, *P < 0.05 vs sham, ^#^ *P* < 0.05 vs MI, β-actin served as a loading control); (d) LDH level in cardiac tissue was determined by LDH assay (one-way ANOVA, *n* = 6, *P < 0.05 vs sham, ^#^ *P* < 0.05 vs MI); and (e) IL-1β and IL-18 levels were determined by ELISA (one-way ANOVA, *n* = 6, *P < 0.05 vs sham, ^#^ *P* < 0.05 vs MI).

Masson-Trichrome staining and RT-qPCR revealed that compared to the sham-operated mice, mice subjected to MI surgery for a duration of 4 weeks exhibited a noticeable MI scar, disordered myocardial fibers and cardiac fibrosis, along with increased expression levels of α-SMA and collagen I, the well-established fibroblast marker for the differentiation of fibroblast to myofibroblast and a marker for the accumulation of extracellular matrix proteins, which were not affected by the intramyocardial delivery of lentivirus-encoded scramble sequence. However, the knockdown of the RIP3 significantly alleviated the disturbance in myocardial fiber arrangement and reduced the mRNA levels of α-SMA and collagen I as well as the formation of fibrosis. These findings indicated that RIP3 knockdown mitigated remodeling of cardiac structure caused by MI (Figure 5a and b).

### 3.5 RIP3 knockdown reduced cardiac tissue inflammation post-MI

Chronic inflammation is known to play a key role in progressive adverse cardiac remodeling after MI. RIP3, a key regulator of programmed necrosis, has been reported to be linked to inflammatory cell death. We previously showed that there is progressive upregulation of RIP3 in the infarcted border zone of the hearts of MI mice, along with increased cardiomyocyte necrosis. Knockdown of RIP3 improved the death of necrotic cardiomyocytes. Herein, we confirmed myocardial necrosis, as evidenced by a significant increase in the release of LDH after MI. Moreover, we observed increased levels of IL-1β and IL-18 in the heart tissue of MI mice as compared with sham mice. In comparison with the MI mice, the levels of LDH, IL-1β, and IL-18 were not affected by the intramyocardial delivery of scramble shRNA, but were significantly decreased by knocking down of RIP3 in the heart tissue. These results indicated that the deletion of RIP3 mitigated myocardial necrosis and inflammatory myocardial injury after MI (Figure 5c and d).

### 3.6 RIP3 knockdown exerts anti-inflammatory effect *via* modulating nuclear factor kappa-B (NF-κB)/NLRP3 pathway

**Figure 6:**
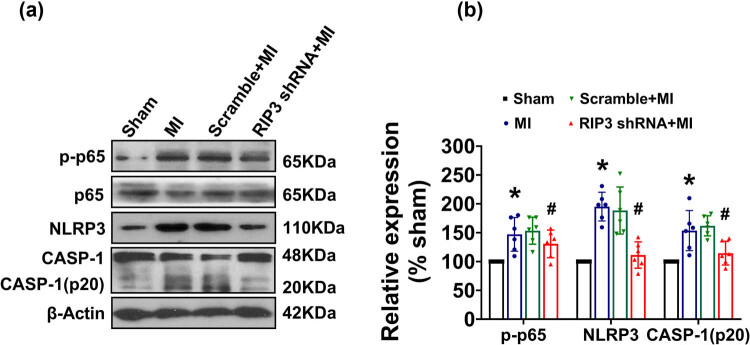
RIP3 knockdown inhibited the activation of the NF-κB/NLRP3 pathway. The total protein expression and phosphorylated level of p65, and NLRP3, caspase1 p20(CASP1(p20)) in heart tissues were examined by western blotting, respectively: (a) representative blots depicting total and phosphorylated proteins and (b) the analyzed results of p-p65 level, NLRP3, and CASP1(p20) level (one-way ANOVA, *n* = 6, *P < 0.05 vs sham. ^*#*^ *P <* 0.05 vs MI).

Given that IL-1β and IL-18 are important effector proteins of the NF-κB/NLRP3 signaling pathway, we evaluated the protein expression of the NF-κB/NLRP3 pathway. Figure 6 showed that there was a significantly increased phosphorylation of p65 in heart tissue of MI mice compared to sham mice. However, the phosphorylation level of p65 was not affected by the intramyocardial delivery of scramble shRNA, but was significantly attenuated by the deletion of RIP3 when compared to MI mice. Additionally, we observed a significant increase in the expression of NLRP3 and cleaved caspase-1 (p20) in the hearts of MI mice compared to sham mice. Once again, the expression of NLRP3 and cleaved caspase-1 (p20) was not affected by scramble shRNA, but was markedly reduced by the deletion of RIP3 in the mouse hearts.

## 4 Discussion

The main findings of this study are as follows: (a) mice with MI for 4 weeks exhibited impaired LV myocardial mechanics, as evidenced by a significant decrease in radial and circumferential strain and strain rates of all segments at the papillary muscle level during systole and early diastole. (b) Knockdown of RIP3 increased radial and circumferential strain and strain rates during systole and diastole and mitigated the impairment of LV myocardial mechanics and cardiac dysfunction induced by ischemic injury. (c) The percentage decrease of SRrad-S and SRcir-S showed a strong negative correlation with LVEF, while positive correlation with LVIDs both in MI mice and mice with RIP3 knockdown. (d) The decreased cardiac tissue inflammation by the deletion of RIP3 may contribute to the amelioration of LV myocardial mechanics and dysfunction post-MI.

Growing evidence supports the importance of myocardial mechanic alteration in adverse cardiac remodeling post-MI [26,27,28]. However, assessing regional LV mechanics remains challenging. Conventional echocardiography is commonly used to evaluate metrics of cardiac function, such as EF and LVFS. While these metrics are valuable in assessing the overall impact of ischemic injury on global myocardial function, they do not capture regional differences in myocardial contractility. Two-dimensional speckle tracking technology, which is independent of angle and surrounding tissue movement, allows for the quantification of myocardial multi-dimensional strain and provides a more accurate reflection of myocardial mechanics. It enables a detailed assessment of both global and regional LV function in the longitudinal, circumferential, and radial directions [20,21,22,23,24]. Several studies have shown significant reductions in regional strain of ischemic LVs compared to healthy hearts. The reported strain difference between the infarcted and remote myocardium is related to infarct expansion from infarct zones to remote border zones [29,30,31,32]. Assessing segmental strain and strain rate has also been shown to be helpful in predicting adverse remodeling in ischemic cardiomyopathy and provides important prognostic clues for predicting heart failure in patients with postinfarction [33]. Using speckle tracking technology, we observed segmental wall motion abnormalities in the LV, along with thinning of the LV wall, enlarged LVID, increased cardiac fibrosis and necrosis, as well as cardiac systolic and diastolic dysfunctions in mice subjected to MI for 4 weeks. The radial and circumferential strain and strain rates during systole and diastole in all segments of the LV wall in MI mice were significantly lower, particularly in the anterior wall and lateral wall where severe injury occurred due to the occlusion of the LAD coronary artery. These results indicated that local myocardial ischemia resulting from anterior MI led to impaired regional myocardial mechanics. This impairment can potentially place an increased burden on the non-infarcted myocardium, thereby affecting the mechanics of all myocardial segments and ultimately resulting in a decrease in overall systolic and diastolic motion and function of the LV.

Myocardial inflammatory necrosis plays a key role in adverse cardiac remodeling and impaired cardiac mechanics after MI [3,4,5]. It has been reported that shortly after an acute MI, alterations in the heart’s structure are observed, which is characterized by the infarcted area undergoing necrosis, inflammation, and replacement fibrosis. These processes result in local thinning, scar formation, and increased stiffness of the ventricular wall, along with loss or paradoxical motion of myocardial segments. These alterations may potentially exacerbate the expansion of the infarct area, ultimately resulting in decreased cardiac systolic and diastolic functions [26,27,28]. We previously demonstrated that RIP3-mediated necroptosis contributes to the loss of functional cardiomyocytes and cardiac dysfunction following MI. Knockdown of RIP3 has been shown to increase the survival of ischemic cardiomyocytes, reduce the size of the infarction, and notably improve MI-induced cardiac dysfunction [13]. In the current study, we further investigated the role of inflammatory signaling pathways in the protective effect of RIP3 knockdown against MI. We found that IL-18 and IL-1β-driven inflammation were activated after MI, but this activation was significantly inhibited by RIP3 knockdown. It is well established that IL-18 and IL-1β-driven inflammation play a crucial role in the development of adverse cardiac remodeling by regulating extracellular matrix metabolism and fibroblast function [16,17]. Therefore, it is likely that both the reduction in cardiomyocyte necrosis and subsequent inflammation achieved by knocking down RIP3 in the ischemic heart contribute to the suppression of adverse cardiac remodeling and ultimately a substantial improvement in cardiac mechanics. Indeed, our findings support this hypothesis. We observed that RIP3 knockdown suppressed MI-induced upregulation of α-SMA and collagen I and alleviated the cardiac fibrosis, resulting in increased radial and circumferential strain and strain rates, not only in the ischemic anterior and lateral walls but also in other remote segments of the LV wall. The percentage decreases in SRrad-S and SRcir-S, during both systole and diastole across various segments in mice with RIP3 knockdown, were significantly lower compared to that observed in MI mice. Importantly, these percentage decreases showed a strong correlation with LVEF and LV end-systolic diameter. These findings provided further evidence that reduced cardiomyocyte necrosis and mitigated inflammatory injury through RIP3 knockdown effectively improved cardiac mechanics and cardiac dysfunction following MI. The percentage decrease in strain rate of the infarcted myocardium could be served as a crucial reference index for evaluating cardiac function.

Inflammasome activation mediated by NLRP3, a member of the nucleotide-binding oligomerization domain-like receptor (NLR) family, plays a pivotal role in inflammatory injury driven by IL-18 and IL-1β. Multiple studies have shown that various microbial or damage-associated molecular patterns could induce the activation of NLRP3 inflammasome. Once activated, NLRP3 inflammasome triggers the formation of mature caspase-1(p20) by proteolytic cleavage of caspase-1, leading to the release of active IL-1β and IL-18 from their pro-forms [34]. Growing evidence suggests that the activation of NLRP3 inflammasome is significantly involved in the pathophysiology of cardiovascular diseases, including atherosclerosis and acute MI [35]. In accordance with these results, we observed higher levels of NLRP3 and caspase-1 (p20) in the hearts of MI mice. Importantly, cardiac-specific knocking down of RIP3 resulted in a decreased expression of NLRP3 and caspase-1 (p20), along with lower levels of IL-1β and IL-18, suggesting that the suppression of the NLRP3 inflammasome by RIP3 knockdown is involved in its major anti-inflammatory action. Consistent with our results, the RIP3-mediated activation of the NLRP3 inflammasome was also observed in caspase-8-deficient dendritic cells treated with LPS and acute kidney injury [36,37]. Furthermore, we detected NF-kB activation along with the upregulation of NLRP3 and caspase-1(p20) following MI, which could be attenuated by RIP3 knockdown. Canonically, the activation of fully functional NLRP3 inflammasome requires two steps: priming and activation. Although the exact molecular mechanisms for NLRP3 activation remain incompletely understood, it has been found that the activation of NF-kB is required for the priming step to produce pro-IL-1β and optimum NLRP3, which is necessary for the subsequent activation of the NLRP3 inflammasome and the release of active IL-1β [34]. Together, these data implicated that RIP3 knockdown has the potential to inhibit myocardial inflammation after MI by targeting the NF-κB/NLRP3 inflammatory signaling pathway. Nevertheless, further studies are needed to elucidate the precise mechanisms linking RIP3 and inflammation mediated by NLRP3 inflammasome.

It is noteworthy that the activity of NF-κB can be induced or enhanced by pro-inflammatory signals and antigen receptors. Studies have demonstrated that activated IL-1β or IL-18 can enhance the activity of NF-κB. Moreover, overactivation of NF-κB can further trigger the transcription of numerous genes encoding pro-inflammatory cytokines such as tumor necrosis factor-α (TNA-α), IL-6, and IL-1β [38,39]. As a crucial mediator of the inflammatory response, the inflammatory cascade mediated by the overactivation of NF-κB has been implicated in various human diseases, including ischemic heart disease, cancer, autoimmune disorders, and viral infections [40,41,42,43,44,45]. Therefore, it is conceivable that, in addition to the RIP3-mediated NF-κB/NLRP3 signaling pathway, the activation of other inflammatory signaling may be involved in the inflammatory mechanisms underlying RIP3 in the context of MI, necessitating further investigation.

## 5 Conclusions

**Figure 7:**
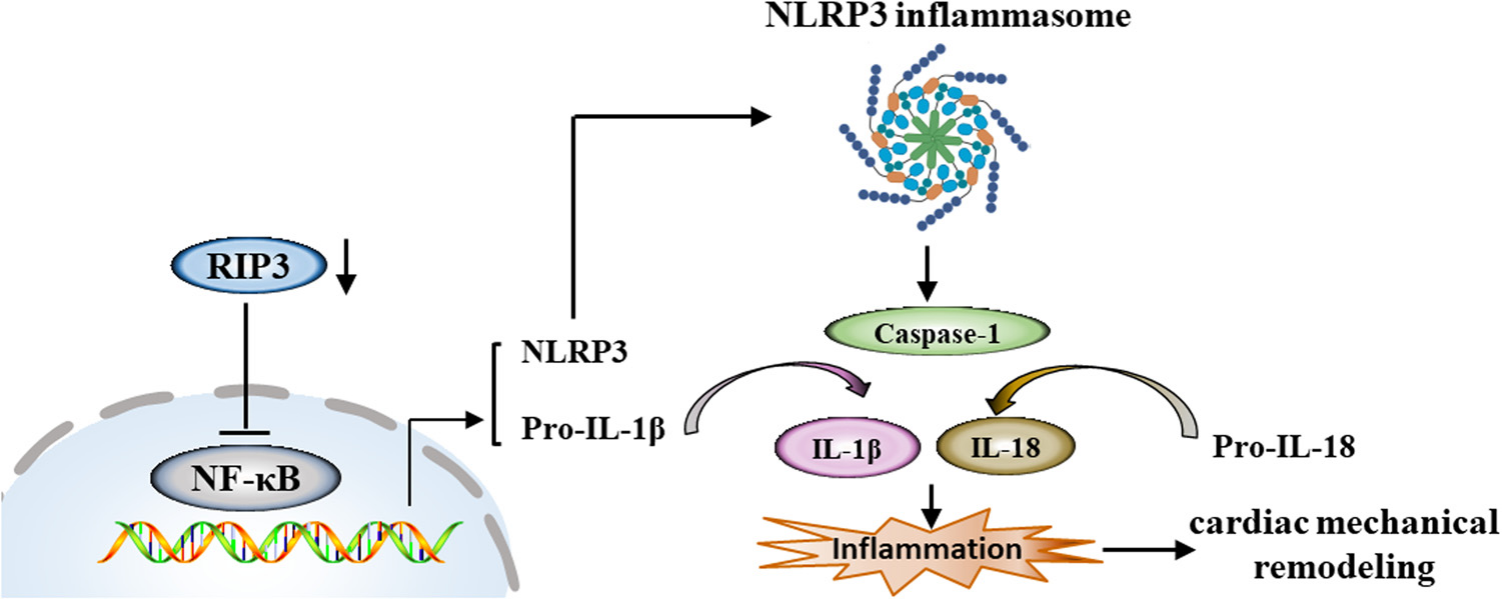
Scheme of downregulation of RIP3 ameliorates the LV mechanics and function after MI *via* modulating the NF-κB/NLRP3 pathway. The downregulation of RIP3 shows potential for preventing cardiac mechanical remodeling and heart dysfunction following MI by inhibiting inflammatory cardiac injury through regulating the NF-κB/NLRP3 signaling pathway.

This study provides additional insight into the changes in LV mechanics following ischemic injury. We demonstrate that impaired myocardial mechanics are not confined to the ischemic segments but also affect non-ischemic segments after a 4 week MI. The degree of LV function impairment is directly correlated with the percentage decrease in myocardial strain and strain rate. The downregulation of RIP3 shows potential for preventing cardiac mechanical remodeling and heart dysfunction following MI by inhibiting inflammatory cardiac injury, possibly through regulating NF-κB/NLRP3 signaling pathway (Figure 7).
